# User-centric innovation strategies for cultural creative products in China’s rural tourism

**DOI:** 10.1371/journal.pone.0319474

**Published:** 2025-04-23

**Authors:** Yuzhe Qi, Qing Ni, Jing Zhang, Jiangyue Han, Hongyan Ren

**Affiliations:** 1 Industrial Design Program, Silla University, Busan, Korea; 2 College of Art and Design, Henan Finance University, Zhengzhou, China; Gazi University Faculty of Engineering: Gazi Universitesi Muhendislik Fakultesi, TÜRKIYE

## Abstract

Cultural creative products play an important role in the revitalization of tourism-oriented rural areas and the preservation of cultural heritage. However, existing products fail to fully meet the diverse needs of consumers and lack a systematic design framework. This study proposes a user-centered framework aimed at guiding the innovation of cultural creative products in China’s rural tourism. During the exploration of user needs, grounded theory was used to identify three core user needs: emotional identification, personalization and uniqueness, and enhancement of quality of life. Using the KANO model, these needs were further categorized into basic needs, performance needs, and excitement needs, clarifying their priorities. In the stage of translating user needs into actionable design elements, the Analytic Hierarchy Process (AHP) was employed to assign weights to each need, and Quality Function Deployment (QFD) theory was used to construct the House of Quality, refining 14 user needs into 9 key design elements. The findings indicate a positive correlation between multifunctional design and ergonomic design, that usability and convenience are closely related to human-machine ergonomics, and that the integration of cultural symbols with emotional design and innovative forms significantly enhances the cultural value and user experience of the products. This study provides a systematic theoretical framework and practical guidance for the innovation of rural cultural creative products, with significant implications for future research and practice.

## 1. Introduction

Rural areas not only possess abundant natural resources and unique cultural heritage but also offer significant social, economic, and ecological benefits [1]. However, with the acceleration of globalization and urbanization, rural regions in China are facing challenges such as population loss [2], economic decline [3], and the erosion of cultural heritage [4]. These issues not only affect the sustainable development of rural areas but also undermine their social and cultural functions. To address these challenges, the Chinese government launched the Rural Revitalization Strategy in 2018, aimed at promoting the prosperity of rural cultural industries [5], upgrading industrial structures, and enhancing social stability and harmony [6]. Against this backdrop, the cultural and creative industries have gradually become a key driver of rural revitalization [7], especially in regions with tourism resources [8]. By transforming the rural areas’ historical culture [9], folk traditions [10], and natural landscapes into marketable products [11], the cultural and creative industries contribute to economic diversification and promote the sustainable development of rural areas [12]. However, with changing social consumption trends, traditional cultural and creative product designs are no longer able to meet the needs of modern consumers. Consumers are not only concerned with cultural identity but also expect these cultural elements to integrate with their personal lifestyles, emotional needs, and the evolving social environment. This shift demands that the design of cultural and creative products focus more on personalization, functionality, and innovation to adapt to the continuously changing market demands.

Existing research in the rural cultural and creative industries has made some progress. For example, Feng proposed integrating the cultural elements of ancient villages into the packaging design of cultural and creative products [13]. However, this research did not fully consider whether these cultural elements could capture the attention and purchasing desires of modern consumers. Wu analyzed rural home products, particularly the cultural value and visual characteristics of tea-picking lamps, and successfully developed locally themed products [14]. However, the design overly focused on cultural expression, neglecting consumer demands for personalization, functionality, and innovation. This demonstrates that existing research on the rural cultural and creative industries has largely concentrated on showcasing cultural elements, while failing to align with market needs, particularly the ever-evolving consumer demands [15]. With the diversification of market demand and the rise of personalized user needs, product development must be driven by user requirements in order to truly achieve market competitiveness [16]. Existing research indicates that a deep understanding of user needs not only helps improve product market acceptance but also stimulates innovation in product design [17]. Therefore, there is an urgent need to shift towards a design model that places greater emphasis on user needs to achieve better market adaptability.

As consumers increasingly demand personalization, emotional fulfillment, and functionality, rural cultural and creative products urgently require more precise user demand analysis to enhance market adaptability [18]. Failing to respond to these shifting demands in a timely manner could lead to missed opportunities for both cultural and economic value enhancement, thereby affecting the success of the Rural Revitalization Strategy [19]. Therefore, the core objective of this study is to enhance the market adaptability of rural cultural and creative products and support the successful implementation of the Rural Revitalization Strategy through innovative design methods combined with accurate user demand analysis. To this end, this study will focus on the following two core questions:

How can user needs for rural cultural and creative products be precisely identified?

How can identified user needs be translated into diverse and innovative product designs?

To achieve this goal, this study will systematically explore user needs and examine how these needs can be effectively incorporated into the design elements of rural cultural and creative products. Propose innovative design strategies through research to provide a theoretical foundation and practical guidance for the development and growth of the rural cultural industry. This approach not only captures consumer demands more accurately but also transforms these needs into creative and market-attractive product design elements. This process holds significant theoretical and practical implications for advancing the rural cultural and creative industries.

This research employs a combined qualitative and quantitative approach, incorporating Grounded Theory, the KANO model, AHP (Analytic Hierarchy Process), and QFD (Quality Function Deployment) for user demand analysis and design element matching. This integrated approach enables an in-depth understanding of user needs, with particular advantages in capturing potential and dynamic demands. Compared to traditional methods, such as single-user surveys [20], personas [21], participatory design [22], and prototype testing [23], this study offers a more comprehensive and precise insight into user needs. This insight will effectively guide the design and development of rural cultural and creative products, thereby enhancing their market competitiveness. Grounded Theory and KANO help uncover and analyze deep emotional needs, while AHP and QFD provide systematic matching of user needs and design elements, ensuring a high degree of alignment between design and market demands.

This study not only fills the gap in the current lack of user-demand-oriented research in the development of rural cultural and creative products but also proposes a new approach that closely integrates user needs with creative design, thereby offering a new development path for rural revitalization. Specifically, this study systematically examines user needs and explores how to fully incorporate these needs into the development process of cultural and creative products. It also proposes innovative design strategies that will help enhance the market competitiveness of rural cultural and creative products, thereby driving economic growth and cultural heritage preservation in rural areas. This research provides valuable insights for both theoretical studies and practical applications in related fields.

## 2. Theoretical examination

In the theoretical examination section, we will explore the relevant theories in the rural cultural creative product development process from two core perspectives, based on the two research directions proposed in Chapter One. The first aspect is the exploration of user needs, which is currently lacking in rural cultural creative product development. Therefore, understanding the theoretical framework of user needs is crucial. The second aspect involves examining the integration of user needs with design elements, focusing on how relevant theories can effectively translate user needs into design elements. We will then explore how these theories can be integrated and explain why they can better support this study’s investigation into user needs and their transformation into design elements.

### 2.1 Theory of user needs exploration

#### 2.1.1 User-driven innovation theory.

User-Driven Innovation (UDI) differs from traditional “top-down” innovation approaches by positing that users, particularly “lead users,” they can directly influence the development of products and services through their needs and experiences [24]. In his research, E. Von Hippel points out that users, through their actual use of a product, can identify needs and issues that designers may not have considered during product development. This feedback can then provide designers with new inspiration and direction for product development [25]. In other words, users are not only the final consumers of products but also a primary source of innovation. Therefore, examining users’ expectations and needs is crucial. Research has also found that this user feedback can reveal hidden needs, thereby better guiding product design and improvement, making products more adaptable to the market, and increasing user satisfaction [26].

The core of UDI theory lies in using user feedback (user experiences) as the foundation for designing and improving products. This approach effectively integrates users’ actual experiences and needs into the innovation process, thereby enhancing product market adaptability and user satisfaction. This theory is in line with the aim of this study, as a user-driven approach ensures that the development of rural cultural creative products is more attuned to actual user needs, thereby improving both product market competitiveness and user experience. Although user feedback is crucial, it is important to note that not all users can provide valuable innovative ideas. Researchers need to conduct detailed analysis to identify true “lead users,” which requires significant time and effort. To address this issue, this study employs grounded theory to progressively focus and achieve theoretical saturation, refining core needs from actual user data in a bottom-up manner.

#### 2.1.2 Overview of grounded theory.

Grounded Theory is a qualitative research method that, unlike traditional approaches, focuses on deriving theories “grounded” in actual data rather than starting from pre-existing hypotheses [27]. Therefore, Grounded Theory emphasizes the use of raw data obtained from experiments. It involves breaking down the collected material, identifying phenomena, conceptualizing these phenomena, and then abstracting the concepts appropriately to refine them into categories [28]. The primary means of data collection in Grounded Theory is through interviews. The data refinement process is typically divided into three main parts [29]:

(1) Open Coding: Researchers analyze the collected data sentence by sentence and segment by segment, extracting keywords, phrases, or concepts. Each extracted concept is assigned an open code, which is a brief label or keyword describing the concept [30].
(2) Axial Coding: Researchers organize these codes through comparison and categorization, identifying their interrelationships and interactions to form higher-level concepts and patterns [31].
(3) Selective Coding: Researchers selectively delve into and analyze core concepts related to the research question to identify the most important factors and relationships, further refining and developing the theory [32].

Grounded Theory is extensively used across various fields in the social sciences [33], it is frequently used as a method to uncover user needs in the product design and development process. This is because the theories generated by Grounded Theory typically have strong practical guidance significance [34]. In this study, the theory helps researchers better understand the complexity and diversity of user needs, thereby guiding designers’ practice and fostering innovation and development in their work [35]. However, grounded theory also has its limitations. The identification and classification of user needs are accomplished through iterative coding and analysis. While this process is thorough, it often lacks structured categorization, meaning researchers cannot accurately subdivide needs at the same level. In product development, not all user needs need to be met or implemented [36]. Therefore, by employing the structured approach of the KANO model, which clearly categorizes user needs, the theory becomes more targeted and efficient.

#### 2.1.3 Introduction to the KANO model theory.

**Fig 1:**
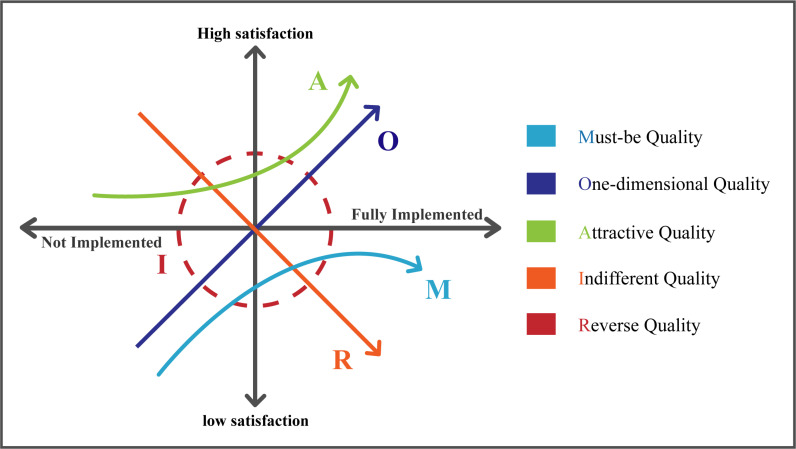
KANO requirements model.

The KANO model is a quality management tool proposed by Norihiko Kano in 1980. This model primarily helps product developers categorize the diverse needs of users [37]. The KANO model focuses on the relationship between user satisfaction and the features of products or services [38]. By categorizing and analyzing the relationship between products and user satisfaction, it helps researchers better understand user needs and provides assistance in developing corresponding product development strategies [39], as shown in Fig 1. By assessing user satisfaction and expectation levels, the KANO model determines the importance of each type of need and categorizes user requirements into five types: basic needs, expected needs, excitement needs, reverse needs, and indifferent needs, as detailed in Table 1. These categorized needs help product developers prioritize which key requirements to address in their products, thereby enhancing market competitiveness. Consequently, this theory is widely applied in various fields, including user experience design [40], product feature exploration [41], interface design [42], service design [43], and sustainable design [44].

**Table 1:** The KANO Model’S Various Demand Indicators And Explanations.

Demand categories	Content	Representative symbols
Must-be Quality	The basic expectations and requirements of users for products or services are essential conditions that must be met.	M
One-dimensional Quality	Explicitly recognized expectations and preferences are the standard expectations of users for products or services.	O
Attractive Quality	The needs that users do not explicitly express but go beyond their expectations are the delight for the product or service provider.	A
Indifferent Quality	The part of product or service features that users do not care about or have no clear attitude towards, even if the product or service has reverse requirements, will not significantly affect user satisfaction.	I
Reverse Quality	The presence or absence of which users do not have a clear attitude towards or affect their satisfaction significantly, and may even reduce satisfaction to some extent.	R

In conclusion, the User-Driven Innovation (UDI) theory provides the theoretical foundation for identifying user needs in this study, while the combination of Grounded Theory and the KANO model offers a scientific framework for transforming these needs into design elements. Grounded Theory helps designers identify latent needs and preferences by analyzing users’ actual demands, thus uncovering the underlying motivations behind user expectations. Meanwhile, the KANO model assists designers in categorizing needs, helping them identify key requirements, and clarifying which needs must be met and which can exceed user expectations to create delight, thereby ensuring these needs are effectively translated into innovative product features. This integration ensures that user needs are efficiently transformed into innovative and practical product designs, providing strong support for the implementation of rural revitalization strategies.

However, in practical application, there are some challenges. First, the coding process in Grounded Theory has a degree of subjectivity, as researchers’ personal preferences and experiences may influence data interpretation, leading to potential biases. To address this, the study incorporated collaboration among multiple researchers, using cross-validation and collective discussion to ensure the objectivity and consistency of the coding process. Second, the classification of needs in the KANO model can become ambiguous or overlapping when faced with complex cultural contexts and diverse user groups, potentially affecting the accuracy of classification results. To resolve this, we refined the categorization of needs through multiple rounds of focus group discussions and data comparisons, ensuring the comprehensiveness and accuracy of the identified needs. By employing these comprehensive methods, the study enhanced the accuracy and innovation of user needs transformation, providing solid theoretical support and practical guidance for the successful implementation of rural revitalization strategies.

### 2.2 Integration of user needs with design elements

#### 2.2.1 Overview of AHP theory.

The AHP (Analytic Hierarchy Process) is a multi-criteria decision-making method developed by Thomas L. Saaty [45], it involves decomposing a problem into a hierarchical structure and comparing and weighing factors at different levels to determine the best decision [46]. The core concept of AHP is to divide a decision problem into a multi-level structure, including the goal level, criteria level, and alternatives level [47]. In this hierarchy, the goal level represents the ultimate objective or decision outcome, the criteria level includes factors affecting the achievement of the goal, and the alternative level consists of possible solutions or decision options, as detailed in Table 2. It is important to note that in AHP, factors are not compared all at once; instead, they are compared pairwise using relative scales to minimize differences between diverse factors and improve accuracy [48], as shown in Table 3.

**Table 2:** AHP Research process.

Sequence	Content
1	Clarify the decision objectives and the scope of the problem, and decompose the problem into multiple levels.
2	Develop a hierarchical model and define the different levels of objectives, criteria, and alternatives.
3	Perform pairwise comparisons of each criterion to determine their relative importance.
4	Calculate the weight of each criterion in relation to the objective and perform a consistency check.
5	Perform pairwise comparisons of the alternatives to determine their relative importance.
6	Calculate the total score for each alternative and choose the one with the highest score as the final decision.

**Table 3:** AHP Matrix construction model.

_*A=*_	$a_{12}$	$a_{12}$	……	……	$a_{1n}$
	$a_{21}$	$a_{22}$	……	……	$a_{2n}$
	……	……	$a_{ij}$	……	……
	$a_{n1}$	$a_{n2}$	……	……	$a_{nn}$

As mentioned above, while the KANO model effectively categorizes user needs, there are still numerous elements within each category, posing a challenge for researchers in selecting representative needs and translating them into design elements. To address this issue, this study uses AHP to rank different user needs within the same category, providing deeper insights into user preferences and the importance of various design features or functions. By systematically analyzing and weighing various factors, AHP can guide the development of innovative strategies for rural cultural and creative products.

#### 2.2.2 Overview of QFD theory.

Quality Function Deployment (QFD) is a systematic research method commonly used in the product design and development stages [49]. It aims to translate customer needs into specific technical requirements and design parameters, ensuring that product design and development accurately reflect customer expectations and requirements [50]. The essence of QFD lies in using the House of Quality tool to integrate the Voice of the Customer (VOC) throughout all stages of product development, thereby ensuring high-quality product outcomes. The advantage of this research method is that it ensures customer needs are thoroughly considered from the initial stages of product development, enhancing the alignment between the product and customer expectations. As a result, it significantly reduces the likelihood of changes during the design and implementation phases, thereby lowering development costs and time risks. According to R. Ginting’s comprehensive review of the literature, QFD technology has been formally established in Japan’s academia and industry for over 50 years since the 1960s and 1970s. Due to its notable effectiveness in quality management and product development, QFD is now widely adopted [51]. In the study by Buakum, the QFD method was applied to the design of a temperature-controlled medicine bag, successfully transforming user needs into specific technical requirements [52]. Using the “House of Quality” tool, the team mapped core user needs, such as temperature control, convenience, and safety, to corresponding technical requirements (selection of temperature control modules, choice of insulation materials), ensuring that the design could fully meet user expectations. The application of QFD effectively improved the accuracy of need translation, ensured the innovation and practicality of the product design, and reduced subjective bias in the design process. It can be seen that QFD adopts a systematic approach to link different levels of needs with design elements, effectively maintaining the consistency between design elements and customer needs.

The combination of AHP and QFD not only scientifically quantifies key user needs but also effectively translates them into specific design elements. Specifically, the quantitative analysis results from AHP help identify which needs are critical to users, while QFD ensures that these needs are accurately transformed into technical requirements and design parameters, thereby ensuring the scientific accuracy and precision of the design. For the development of rural cultural creative products, by precisely capturing consumers’ deep experiential needs for local culture and translating them into innovative product features, market competitiveness can be effectively enhanced. Furthermore, the integration of AHP and QFD reduces uncertainty in the design process, allowing product development to gain an advantage in the highly competitive market, providing reliable theoretical support for the success of rural cultural creative products.

## 3. Construction of the research theoretical framework

Through the analysis of the relevant theories mentioned above, it is evident that each research theory has its unique advantages and limitations. In this study, to systematically explore user needs for rural cultural creative products and effectively translate these needs into design elements, we have integrated Grounded Theory, the KANO model, AHP, and QFD to construct a comprehensive theoretical framework. This framework aims to ensure that the entire process from the identification of user needs to the conversion into design elements is both scientific and systematic. In the integration of theoretical methods, qualitative data (such as interviews and open-ended questionnaires) can deeply reflect users’ emotions and needs, but lack structure and quantification capability; whereas quantitative data, through numerical measurement of needs, provides priority rankings and weight values, but may not fully capture users’ complex emotions and latent needs. Therefore, this study adopts a phased data collection and analysis approach, combining both qualitative and quantitative methods, ensuring the independence and scientific rigor of the data while providing effective support for each stage of the analysis.

According to the UDI theory, user needs and feedback are considered core steps in the research. To gain a deeper understanding of the needs of rural residents, cultural practitioners, and tourists, the study will collect raw data through semi-structured interviews. This data will be processed through the three stages of open coding, axial coding, and selective coding in Grounded Theory to extract the core needs of users. Next, the KANO model will be used to categorize the needs, dividing them into basic, expected, and exciting needs. This process helps clarify the types of needs and their importance to users, providing theoretical support for subsequent prioritization. To ensure clear prioritization of needs, AHP will be employed to conduct a quantitative analysis, providing a scientific basis for design decisions. The results of the AHP analysis will help identify the most critical needs for users, and design decisions will be made based on their weights and priorities. Building on this, the QFD (Quality Function Deployment) tool will be used to translate user needs into specific technical requirements and design parameters, ensuring that the final product design accurately reflects user expectations. For example, in a rural handicraft design project, users expressed a strong preference for traditional woodcarving craftsmanship. In the “House of Quality” of QFD, this need will be translated into a design requirement, such as the application of traditional woodcarving techniques on the wooden casing and ensuring that the carved patterns reflect the local cultural characteristics.

**Fig 2:**
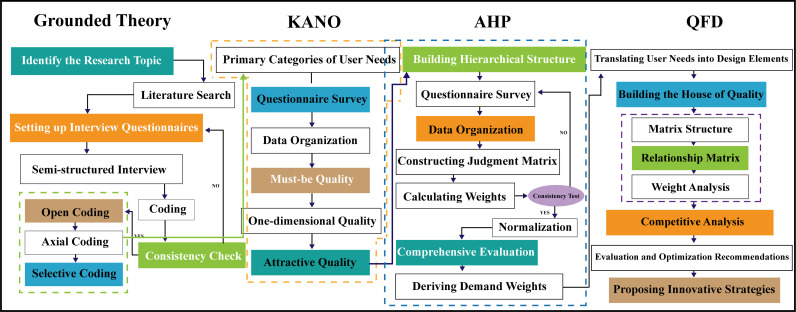
Research process diagram.

Overall, through the organic combination of Grounded Theory, the KANO model, AHP, and QFD, this study ensures that every stage, from need identification to design element translation, is supported by solid theory and data, thereby improving the market competitiveness and user satisfaction of the product. The detailed research process is shown in Fig 2.

## 4. Experimental investigation

### 4.1 Exploration of user needs

#### 4.1.1 Data collection.

Use grounded theory to obtain the most original information from users and process this information.In grounded theory research, interviews are the most frequently used method for data collection [53], particularly suited for exploratory research. Currently, there are three main types of interviews: structured [54], semi-structured [55], and unstructured [56]. To gain a deeper understanding of the complex and varied user needs, this study has chosen to use semi-structured interviews to collect foundational data on user requirements. This is because semi-structured interviews allow researchers to use open-ended questions in the interview guide, which encourages respondents to elaborate on their needs in detail. This approach helps researchers gain a comprehensive understanding of respondents’ perspectives, experiences, and opinions, and uncover potential issues and needs during the interview process [57].

To ensure high credibility of the interview data, the interview guide will be designed based on consumers’ experiences and needs related to cultural and creative products [58]. The interview guide follows the basic principle of starting with easier questions and gradually moving to more difficult ones [59]. The interview questions cover user demographics, needs for cultural and creative products, usage experience, cultural and innovative aspects, emotional connection and sense of belonging, as well as product improvements and suggestions. Some of the interview questions are shown in Table 4.

**Table 4:** Semi-Structured Interview Questions.

Number	Interview questions
1	What is your age, occupation, and educational background?
2	Do you buy local cultural and creative products when you travel in the countryside? If so, what types of products do you usually buy?
3	What kind of cultural and creative products do you think best represent the cultural characteristics of a rural tourist attraction?
4	What do you look for most when purchasing rural cultural and creative products?
5	Have you ever been dissatisfied with a cultural product you purchased? What caused the dissatisfaction?
6	How do you understand the expression of “rural culture” in cultural and creative products?

When selecting participants, this study considered the diversity of rural cultural products and the broadness of the target market. Therefore, participants from different age groups and regions were chosen to ensure that the data represents various user needs and cultural backgrounds. The regional differences reflect the differentiated cultural consumption needs between urban and rural areas, especially in the central and eastern regions of China, where there are certain differences in the ways cultural heritage is passed down and innovation is carried out between urban and rural areas [60]. As a result, selecting participants from this region can better reflect the broader trends in cultural demand. This approach ensures the diversity and representativeness of the sample, and better captures the overall market demand for rural cultural and creative products.

Before the interviews, the research purpose and interview content were explained in detail to the participants, and informed consent was obtained from all participants. All participants were fully informed of the purpose, process, and their rights regarding participation in the study. This study has also received ethical approval from the Human Rights Research Ethics Committee of the College of Design at Silla University, in accordance with relevant ethical regulations. During the interviews, participants were gradually guided to explore their experiences, starting from their general impressions. Since the participants were geographically dispersed, the study was conducted through online interviews. Online interviews, as a convenient method, overcome geographic limitations and are suitable for covering a wide range of respondents [61]. Although online interviews lack the immediate interaction found in face-to-face interviews, which may limit the depth of information gathered, the flexibility and efficiency of online interviews make them a suitable method for this study, especially considering the geographical distribution and sample size. According to the principles of grounded theory, sample size in qualitative research is not determined by quantity but by data saturation. When no new significant information emerges during the interviews, data saturation is considered to have been reached [62]. After validating the saturation, the first 12 interviewees were selected as the primary subjects for this study. The sample included 7 males and 5 females, with ages ranging from 18 to 52 years. The interviews were conducted from December 8, 2023, to February 3, 2024, primarily with participants from central and eastern China. Users participating in the study completed the appropriate learning tasks and simulation exercises according to the teaching requirements and filled out an anonymous questionnaire at the end of the course. At the same time, the research team obtained consent from some users and conducted individual interviews. Following the requirements of qualitative research in grounded theory, each participant engaged in in-depth discussions lasting 20–40 minutes based on the interview guide [63]. The collected interview data will be subjected to coding analysis to label, conceptualize, and categorize the raw data. The three-level coding process of grounded theory will be used to further organize, analyze, and summarize the interview data.

#### 4.1.2 Open coding.

Open coding is the first step in information coding, where the primary categories are derived entirely from the interview records, without any subjective interpretation or judgment [64]. The interview records were extracted by team members based on the recorded interviews. To minimize personal subjectivity and ensure coding reliability, all information in the interview data remained open, with multiple researchers independently coding and cross-validating within the team [65]. Independent coding requires the researcher to analyze and label the raw data word by word. The cross-validation session required group members to repeatedly compare the individually organized content, gradually summarize and merge the valid information, and transform and process it into conceptualized and categorical core vocabulary. Through the combing of the original interview text, a total of 368 pieces of valid information were obtained, and by eliminating recurring content and merging semantically similar concepts, 45 initial categories were finally obtained, as shown in Table 5. These initial categories are the direct factors of users’ demand for rural cultural and creative products.

**Table 5:** Open coding categorization.

Raw data statement	Initial category
I am very interested in products that can showcase the charm of traditional craftsmanship.	Traditional craftsmanship
I find products that reflect the historical changes and cultural heritage of the countryside more intriguing.	Cultural heritage
Products with a strong rural flavor and folk characteristics remind me of the traditional rural way of life and folk customs.	Traditional Folklore
The products that resonate with me can enhance my emotional experience and purchasing motivation.	Emotional Resonance
A product with personalized design can embody my unique taste and personality.	Personalized Design
Capable of breaking free from traditional design constraints, presenting innovative and unique design concepts and creative expressions.	Creative Style
Products with rich local characteristics and regional cultural atmosphere are filled with the joy of exploration.	Regional Characteristics
Products with exquisite styling design can bring visual and tactile pleasure.	Styling Design
Limited production or uniquely designed products can better highlight my taste and identity.	Rarity
I prefer products that are easy to operate.	Human-Computer Ergonomics
Products showcasing rural cultural characteristics and local folklore make me feel the inclusiveness and diversity of culture.	Cultural Education
It’s also a good idea to revisit the good times through products	Fond Memories
The non-toxic and eco-friendly features of rural creative products are important considerations for purchase.	Non-toxic Environmental Protection
It would be great if products could bring joy to me and my friends or family, enhancing emotional exchange between us through shared experiences.	Social Interaction
I prefer products with bright colors and harmonious combinations.	Color Coordination
Products with local characteristics and regional cultural atmosphere can showcase the rich and diverse local culture and historical heritage.	Local Cultural Identity
Those exquisitely designed and beautifully styled products can bring visual enjoyment.	Artistic Taste
I enjoy purchasing products that reflect the common values and lifestyles of societal groups.	Cultural Groups
Products that bring a completely new user experience would pleasantly surprise me.	Functional Innovation
Products that reflect hometown nostalgia and sentimental attachment to the homeland are more attractive.	Hometown Sentiment
I believe traditional craftsmanship is an important part of rural culture, adding unique charm and cultural significance to products.	Traditional Crafts
I like to purchase products that convey traditional family values and ethnic spirit.	Traditional Values
For me, the emotional affinity of rural creative products is an important purchasing factor.	Emotional Affinity
I prefer products that are soft to the touch and have a comfortable texture.	Material Comfort
I would prioritize considering whether the materials used in the product are natural.	Natural Materials
I find products that cater to my specific interests and personal pursuits more appealing.	Hobbies and Interests
It’s also important for products to harmoniously coexist with the natural environment and showcasing natural beauty is also nice.	Natural Environment
I tend to prefer products that are well-designed and easy to operate.	Ease of Use
Products that reflect the details of rural life and the rustic lifestyle allow me to experience the simplicity and beauty of the countryside.	Rural Life
It would be nice if it has some historical significance and collectible value.	Collectible Value
It would be great if it could make my life more colorful, allowing me to experience the beauty and joy of life.	Enjoyment of Life
There’s nothing better than having it tailor-made according to my specific needs.	Exclusive Customization
Products that are environmentally friendly and also promote health are a good choice.	Eco-friendly
I believe products that embody traditional craftsmanship, traditional festivals, and traditional customs, and other cultural heritage are more attractive.	Cultural Heritage
Exquisitely designed and of high quality, rural creative products can enhance the quality and comfort of my life.	Exquisite Taste
Products with exquisite craftsmanship and fine workmanship showcase the craftsmanship and professional skills of the makers.	Exquisite Craftsmanship
I find products with artistic elements more appealing.	Artistic Fusion
I would prioritize choosing rural creative products that are highly practical.	Practicality
The products that showcase my pursuit of art and love for beauty are my top priority.	Artistic Taste
A minimalist yet stylish and sophisticated design would better reflect my taste.	Design Style
It would be more appealing if I could personally design and make the products.	DIY
If I can experience the charm and uniqueness of different cultures, it will increase my interest in purchasing.	Cultural Experience
Whether the purchase is convenient and if the product is easy to use.	Convenience
I prefer to choose products that are reliable in quality and durable.	Durability
I hope a product can have multiple functions, not only to meet basic needs but also to provide additional functions and services.	Versatility

#### 4.1.3 Axial coding.

Axial coding is the process of organizing and categorizing the initial categories derived from open coding to form more abstract and generalized themes or categories [66]. At this stage, the relationships between independent categories were not yet clear. Therefore, in this study, the 45 initial categories were reverted to the original textual data for analysis of their interrelationships. Based on this, the research team conducted repeated comparisons and clustering analyses, ultimately grouping the 45 initial categories into 14 main themes. The categorization process followed these steps: First, the 45 initial categories were preliminarily grouped based on their semantic similarities. Then, through analyzing the relationships between the categories, core concepts and their underlying connections were identified, leading to the final determination of 14 themes, as shown in Table 6. These themes encompass the 45 initial categories and form the theoretical framework of the study regarding user needs, representing the key factors influencing users’ demand for rural cultural and creative products. All categorization processes were independently reviewed and discussed by the team members to ensure the rationality and consistency of the classification.

**Table 6:** Main category and core category coding summarization.

Core Categories	Main Categories	Initial Categories
Emotional Identity A1	Cultural Inheritance B1	Traditional Crafts C1
		Historical Culture C2
		Folk Traditions C3
		Regional Characteristics C4
		Cultural Education C5
	Regional Belonging B2	Hometown Sentiment C6
		Rural Life C7
		Local Cultural Identity C8
	Community Identity B3	Cultural Groups C9
		Hobbies and Interests C10
	Cultural Reverence B4	Traditional Values C11
		Cultural Heritage C12
	Emotional Experience B5	Emotional Resonance C13
		Fond Memories C14
		Lifestyle Enjoyment C15
		Cultural Experience C16
Personalization and Uniqueness A2	Customization Service B6	Personalized Design C17
		Customized Tailoring C18
		Do-It-Yourself (DIY) C19
	Unique Design B7	Creative Style C20
		Artistic Fusion C21
		Styling Design C22
		Functional Innovation C23
	Handcrafted Quality B8	Artistic Taste C24
		Traditional Crafts C25
		Exquisite Craftsmanship C26
	Limited Edition Product B9	Collectible Value C27
		Rarity C28
Enhancement of Life Quality A3	Comfortable Experience B10	Human-Machine Ergonomics C29
		Material Comfort C30
		Usability C31
		Emotional Affinity C32
	Quality Living B11	Natural Environment C33
		Social Interaction C34
		Delicate Taste C35
	Health and Environmental Protection B12	Non-toxic Eco-friendliness C36
		Natural Materials C37
		Ecological Friendliness C38
	Aesthetic Enjoyment B13	Design Style C39
		Color Coordination C40
		Artistic Taste C41
	Functional Utility B14	Convenience C42
		Practicality C43
		Durability C44
		Versatility C45

#### 4.1.4 Selective coding.

Selective coding is the process of integrating main categories by refining core categories through axial coding to initially form a theoretical model [67]. Firstly, the logical connections and relationships between the main categories are deeply analyzed based on the interview data, in order to distill core categories that can play a guiding role. Secondly, through multi-level coding and in-depth analysis, these core categories are linked with other categories to form a theoretical model. Based on the results of axial coding, user needs can be further categorized into three aspects: emotional identification, personalization and uniqueness, and enhancement of life quality, as detailed in Table 6. Emotional identification refers to the degree to which users connect with the emotions expressed by the product. This includes users’ emotional connection to the product, their sense of identification with it, and their agreement with the values conveyed by the product. Personalization and uniqueness refer to the customization and innovation of the product to meet the individual needs of different users, as well as the product’s unique features and qualities. This includes the extent to which the product meets personalized needs, as well as its differences and uniqueness in design, functionality, or experience compared to other products. Enhancement of life quality refers to the improvement of users’ quality of life and experiences through rural cultural and creative products. This includes the comfort, convenience, and practicality provided by the product, as well as related social and cultural experiences, thereby enhancing users’ quality of life and well-being. Finally, based on the verification procedures of grounded theory, theoretical saturation was reached. Specifically, during the data analysis stage, recurring themes and concepts were encountered multiple times, with increasing frequency across different interviews, and no new information was introduced. For example, the theme of “emotional identification” repeatedly appeared in multiple interviews, each time involving similar user emotional responses and value recognition, without expanding into new dimensions. Therefore, this study concludes that theoretical saturation has been achieved, and further data collection is unnecessary.

### 4.2 User needs classification

#### 4.2.1 Questionnaire design.

Through the exploration of grounded theory, deeper and potential user needs and motivations were uncovered, especially those that users might not explicitly express themselves. However, the generated need information is often unstructured, making it difficult for designers to determine which needs are crucial for user satisfaction. To systematically understand user needs and guide design innovation, the KANO model was used for categorization. Grounded theory refines user needs into three categories: initial categories, main categories, and core categories, which influence each other. The main categories are intermediate factors that bridge the other categories. They represent the fundamental aspects of product or service functions that directly impact customer satisfaction and perception. Therefore, the KANO model primarily categorizes the attributes of the main categories. A KANO questionnaire was developed to survey the 14 user needs identified in the main categories, using a Likert scale format [68]. The questionnaire design employs both positive and negative question formats, with each question offering five response options: “Like,” “Expected,” “Neutral,” “Tolerable,” and “Dislike,” corresponding to scores of 5, 4, 3, 2, and 1, respectively (S1 File). This is used to measure users’ attitudes toward satisfaction and dissatisfaction with a particular need [69], as shown in Table 7.

**Table 7:** KANO Model questionnaire numerical indicators.

Demand indicators	Options	Very satisfied	Deserves it	Doesn’t matter	Barely accepts it	Very dissatisfied
18 requirements (B1-B14)	Provision of the requirement	5	4	3	2	1
	The requirement is not provided	5	4	3	2	1

#### 4.2.2 Questionnaire distribution.

To ensure that the user need data obtained from the KANO model survey has sufficient representativeness and statistical validity, we used the concept of confidence intervals to determine the number of questionnaires to distribute [70].


$$\text{n}=\frac{\text{Z}²\times \text{P}\times （1-\text{P}）}{\text{E}²}$$ (1)

As shown in Formula (1), n is the required sample size; Z is the Z-score corresponding to the chosen confidence level; p is the estimated population proportion; and E is the margin of error, representing the maximum acceptable difference between the estimated proportion and the true proportion. In the field of market research, a 95% confidence level has become the default or standard choice [71]. Thus, this study chose a 95% confidence level, corresponding to a Z value of 1.96. To ensure a conservative estimate of the sample size, we selected p=0.5. This is a commonly used conservative estimate because it results in the largest sample size calculation, covering the worst-case scenario for sample needs. The acceptable margin of error E is set at 0.05, meaning we can tolerate a 5% error range. Substituting these parameters into the sample size calculation Formula 2:


$$\text{n}=\frac{1.96²\times 0.5\times （1-0.5）}{0.05²}=384.16$$ (2)

The calculation results indicate that, at a 95% confidence level and a 5% margin of error, at least 384 valid questionnaires are needed for this design evaluation. Considering potential issues such as invalid questionnaires, we increased the actual number of distributed questionnaires to 450 to further ensure sample adequacy and survey result reliability. To ensure the objectivity and scientific validity of data collection, we used random distribution and issued 450 survey questionnaires to the target population [72]. During the distribution process, we primarily used social media platforms related to rural culture and email lists as our main channels. A total of 428 valid questionnaires were collected, resulting in a 95% response rate. The survey participants were aged between 18 and 48 and had experience purchasing rural cultural products.

#### 4.2.3 Questionnaire organization and analysis.

The collected questionnaire data were organized and summarized using a spreadsheet. According to Table 8, the attributes were classified based on positive and negative responses, resulting in a summary of user needs’ KANO categories, as shown in Table 9. The specific steps for organization are as follows:

**Table 8:** KANO Model questionnaire numerical indicators.

Demand	Not providing this demand					
**Providing this demand**		Like	Should be so	No preference	Tolerable	Don’t like
	Like	Q	A	A	A	O
	Should be so	R	I	I	I	M
	No preference	R	I	I	I	M
	Tolerable	R	I	I	I	M
	Don’t like	R	R	R	R	Q

**Table 9:** Main category user demand segmentation.

Demand	A/%	O/%	M/%	I/%	R/%	Q/%	Classification results
B1	21.35	20.05	38.56	20.04	0	0	M
B2	23.81	12.18	42.36	21.65	0	0	M
B3	21.93	19.56	33.68	22.85	1.82	0.16	M
B4	19.56	35.68	21.32	18.95	4.49	0	O
B5	36.58	22.89	12.35	27.68	0.5	0	A
B6	20.83	34.19	19.57	25.41	0	0	O
B7	18.46	36.57	21.67	22.49	0.63	0.25	O
B8	29.56	32.57	18.42	19.12	0.33	0	O
B9	34.92	25.61	18.34	20.97	0.16	0	A
B10	35.73	20.35	13.24	30.68	0	0	A
B11	28.64	36.87	22.54	11.65	0.3	0	O
B12	21.68	24.63	36.54	16.94	0.21	0	M
B13	24.53	31.89	17.15	26.43	0	0	O
B14	19.62	18.57	40.26	20.84	0.59	0.12	M

(1) Data Recording and Summarization: Positive and negative responses for each need point were recorded separately in the spreadsheet. SPSS software was used to aggregate scores for each option, ensuring that all data for each need point were thoroughly recorded.
(2) Frequency Statistics: Frequency statistics help understand user preferences for each need point across different options. The frequency of each option for positive and negative questions was counted in the 428 valid questionnaires.
(3) Kano Classification Mapping: Based on the scores for each need point on positive and negative questions, the combinations of positive and negative responses were mapped to need types. The classified user needs are as follows:

Basic Needs (M): 5 items, including cultural heritage, regional affiliation, community identity, functionality, and environmental health. Improving these basic needs does not enhance user satisfaction, but lacking them will significantly decrease user satisfaction.
Expected Needs (O): 6 items, including cultural reverence, customized service, unique design, craftsmanship quality, quality of life, and aesthetic enjoyment. Expected needs have a linear relationship with user satisfaction; fulfilling these needs increases user satisfaction.
Excitement Needs (A): 3 items, including emotional experience, limited edition products, and comfort experience. In the product market, lack of these needs does not reduce user satisfaction, but fulfilling them greatly enhances user satisfaction.

These classifications provide a solid foundation for further demand analysis and research.

## 5. Extracting design elements

### 5.1 Weight assignment for user needs

Since the user needs categorized by the KANO model are relatively broad and do not account for the interrelationships between needs of the same level, this can result in varying levels of importance for user needs within the same attribute. Therefore, to clarify the relative importance of each need, ensure that the priority reflects the actual situation, and optimize the innovation strategy for cultural products, it is necessary to calculate the weights of the categorized user needs. This step is also crucial for implementing QFD to translate user needs into design elements.

### 5.2 Constructing a hierarchical model

**Fig 3:**
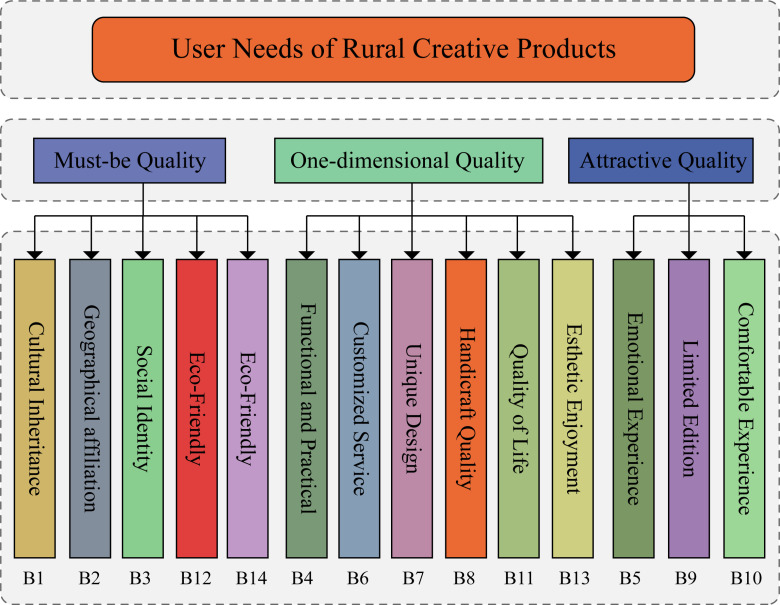
Hierarchical model construction.

Combining the user needs categorized by the KANO model, a hierarchical model was constructed with the user needs for rural cultural products set as the goal layer. The basic needs, expected needs, and excitement needs of users were set as the criteria layer, and the user needs under each attribute were extended to the sub-criteria layer, as shown in Fig 3.

The core of the AHP method lies in evaluating the relative importance of different criteria and alternatives through expert judgment [73]. Therefore, the selection of the expert panel must be relevant and professionally authoritative. The experts chosen for this study all come from fields related to rural cultural and creative products, ensuring that they can provide objective evaluations based on their in-depth expertise during the AHP evaluation process. Additionally, the members of the expert panel come from diverse backgrounds and enjoy high professional reputation, guaranteeing a comprehensive understanding of user needs from multiple perspectives. Specifically, they include:

3 cultural product designers with extensive experience and deep understanding of user needs in the cultural creative sector.
3 rural development scholars with academic backgrounds in the integration of rural development and cultural creativity.
2 government tourism officials responsible for formulating and implementing cultural tourism policies.
1 cultural product collector with practical experience in market demand for cultural products.

These selections are intended to ensure that the judgments relied upon in the AHP analysis are broadly representative and professionally authoritative. To ensure the scientific application of the AHP method and the reliability of the results, a small-scale pilot test was conducted prior to formal data collection. The main purpose of the pilot test was to evaluate the clarity and operability of the questionnaire content, and necessary revisions were made based on feedback [74]. During the pilot test, experts and users pointed out that some question formulations were unclear or could lead to ambiguities, so we optimized certain questions to make them clearer. In the formal AHP survey, each expert was required to use the evaluation scale provided in Table 10 to perform pairwise comparisons of the relative importance of each criterion and sub-criterion based on their professional judgment. The scale ranges from 1 to 9, where 1 indicates equal importance between two factors, and 9 indicates that one factor is of extreme importance compared to the other. To minimize potential biases in expert judgment, each expert was required to complete the questionnaire independently to avoid socialization biases that may arise from group discussions. Furthermore, the research team provided thorough guidance and support throughout the scoring process to ensure consistency and accuracy of the data, thereby minimizing potential judgment biases.

**Table 10:** Evaluation indicators.

Scale	Meaning of representation
1	When comparing two factors, they have equal importance
3	When comparing two factors, the first factor is slightly more important than the second factor
5	When comparing two factors, the first factor is significantly more important than the second factor
7	When comparing two factors, the first factor is strongly more important than the second factor
9	When comparing two factors, the first factor is extremely more important than the second factor
2, 4, 6, 8	The middle value for judging two adjacent factors is referred to as the “intermediate value
The reciprocal of the values mentioned above	When comparing two factors in reverse, it is the reciprocal of the original comparison value

### 5.3 Weight calculation

After collecting all the pairwise comparison scores from experts for the criteria and sub-criteria, the data were compiled to form a judgment matrix. The weight vector of the matrix was then calculated using the geometric mean method. The geometric mean is the nth root of the product of all the variable values. Next, the values were normalized to derive the weight vector. The detailed calculation process is as follows:

1) Normalize the matrix using the following formula (3):

$$\bar{{\text{a}}_{\text{ij}}}={\text{a}}_{\text{ij}}/\overset{\text{n}}{\underset{\text{i}=1}{\sum }}{\text{a}}_{\text{ij}}\left(\text{i}.\text{j}=1,2,\ldots ..\text{n}\right)$$ (3)

Where $a_{ij}$ represents the data in the i-th row and j-th column of judgment matrix A, and $a_{ij}$represents the data in the i-th row and j-th column of the normalized matrix.

2) Sum the elements within the matrix. Calculated as (4):

$$\bar{{\text{w}}_{\text{i}}}=\overset{\text{n}}{\underset{\text{j}=1}{\sum }}\bar{{\text{a}}_{\text{ij}}}\left(\text{i}.\text{j}=1,2,\ldots ..\text{n}\right)$$ (4)

3) Implement normalization for $\bar{w_{i}}$ in the above formula.Calculated as (5):

$${\text{w}}_{\text{i}}=\bar{{\text{w}}_{\text{i}}}/\overset{\text{n}}{\underset{\text{i}=1}{\sum }}\bar{{\text{w}}_{\text{i}}}\left(\text{i}=1,2,\ldots ..\text{n}\right)$$ (5)

Where $w_{i}$represents the weight of the i-th criterion.

4) Compute the largest eigenvalue of the judgment matrix A. Calculated as (6):

$${\text{λ}}_{\text{max}}=\frac{1}{\text{n}}\overset{\text{n}}{\underset{\text{i}=1}{\sum }}\frac{{\left(\text{Aw}\right)}_{\text{i}}}{{\text{w}}_{\text{i}}}$$ (6)

Where n is the order of the matrix, A is the judgment matrix, $w_{i}$is the weight of the ith indicator. ${\text{λ}}_{max}$ is the maximum eigenvalue of the judgment matrix A [75].

5) Consistency checking involves testing the vector and eigenvalue obtained earlier for consistency. If the matrix passes the test, it indicates that the judgment matrix is constructed reasonably and has interpretive value. Let CI represent the Consistency Index. Calculated as (7,8):

$$\text{CI}=\frac{{\text{λ}}_{\text{max}}-\text{n}}{\text{n}-1}$$ (7)

CR=CI/RI (8)

By using the n value, the RI value can be obtained, as shown in Table 11. This allows for the calculation of the Consistency Ratio (CR). The test is considered satisfactory if CR < 0.1.

**Table 11:** RI indicator.

N	1	2	3	4	5	6	7	8	9	10	11
RI	0	0	0.58	0.90	1.12	1.24	1.32	1.41	1.45	1.49	1.51

Using the above calculation process, the scores from the participants were organized and summarized to derive the judgment matrices and weight values for the criteria and sub-criteria layers, as shown in Tables 12–15. The CR values for all groups were calculated to be less than 0.1, indicating that the consistency check was passed [76].

**Table 12:** Assessment of criteria layer demand weight.

	M	O	A	CR	$w_{i}$
M	1.0	1.4	2.0	0.001	0.45
O	0.7	1.0	1.3		0.31
A	0.5	0.8	1.0		0.24

**Table 13:** Assessment of basic needs weight.

	B1	B2	B3	B12	B14	CR	$w_{i}$
B1	1.0	2.0	3.3	1.3	0.7	0.001	0.25
B2	0.5	1.0	1.4	0.7	0.3		0.12
B3	0.3	0.7	1.0	0.4	0.2		0.08
B12	0.8	1.5	2.5	1.0	0.6		0.20
B14	1.5	3.0	5.0	1.7	1.0		0.35

**Table 14:** One-dimensional quality weight assessment.

	B4	B6	B7	B8	B11	B13	CR	$w_{i}$
B4	1.0	2.0	0.8	0.7	1.3	0.6	0.003	0.15
B6	0.5	1.0	0.4	0.3	0.7	0.3		0.08
B7	1.3	2.5	1.0	0.8	2.0	0.8		0.19
B8	1.5	3.0	1.2	1.0	2.0	0.9		0.22
B11	0.8	1.5	0.5	0.5	1.0	0.4		0.11
B13	1.7	3.5	1.3	1.1	2.4	1.0		0.25

**Table 15:** Assessment of attractive quality weight.

	B5	B9	B10	CR	$w_{i}$
B5	1.0	2.0	1.4	0.001	0.45
B9	0.5	1.0	0.7		0.22
B10	0.7	1.5	1.0		0.33

By multiplying the weights of the sub-criteria layer by the weights of their corresponding criteria, the total weights of the sub-criteria within the hierarchical structure were calculated, as shown in Table 16. The final ranking results provide a clear priority reference for the development and promotion of rural cultural products, ensuring that each level of needs is scientifically and reasonably considered. This also provides a comprehensive weight reference for the subsequent extraction of design elements.

**Table 16:** Weighting of indicators for the integrated evaluation.

KANO Attributes	$w_{i}$	Detailed requirements	$w_{i}$	Comprehensive$w_{i}$	Sorting
M	0.45	B1	0.25	0.113	2
		B2	0.12	0.054	9
		B3	0.08	0.036	12
		B12	0.20	0.090	4
		B14	0.35	0.158	1
O	0.31	B4	0.15	0.047	11
		B6	0.08	0.025	14
		B7	0.19	0.059	8
		B8	0.22	0.068	7
		B11	0.11	0.034	13
		B13	0.25	0.078	6
A	0.24	B5	0.45	0.108	3
		B9	0.22	0.053	10
		B10	0.33	0.079	5

### 5.4 Design feature transformation

#### 5.4.1 Constructing the house of quality.

Based on the importance of user needs, the research team converted these needs into specific design elements and features. In this process, the team collaborated with relevant cultural product designers and employed focus group methods to discuss and determine product design characteristics based on core user needs. Since different design functions in product development may simultaneously address multiple needs, and some needs may require the implementation of multiple design functions concurrently [77], the research team used a three-level classification system derived from grounded theory to guide the group members in discussing design elements for each category, considering functional requirements and the cultural background of the target users. This approach helped identify the core design features. For example, the need for emotional resonance was transformed into the design feature of “using soft colors.” This transformation follows a path from abstract to concrete: emotional resonance is a psychological and emotional need, while soft colors, as a design language, can influence users’ emotions through color choices, thereby stimulating emotional resonance. Ultimately, through the focus group discussions, nine core design elements were identified, see Table 17. These elements not only meet user needs but also effectively integrate cultural heritage with modern design requirements.

**Table 17:** Introduction to design elements and design measures.

Code	Design Element	Design Measures
D1	Extraction of Cultural Symbols	Collect local traditional patterns and integrate them with modern design elements in the product.
D2	Preservation of Ancient Techniques	Maintain ancient techniques, reflecting traditional methods in the product.
D3	Customization Options	Offer customization options (color, material, pattern).
D4	Ergonomic Design	Conduct ergonomic analysis during the design phase to ensure the product meets comfort and size requirements.
D5	Design Elements for Emotional Resonance	Use soft, natural colors to evoke emotional resonance in users.
D6	Innovative Form	Experiment with novel product forms to break conventional design frameworks.
D7	Usability and Convenience	Optimize the product’s operation process for ease of use and reduced user learning costs.
D8	Multifunctional Design	Integrate multiple functions into the product to meet diverse user needs.
D9	Use of Eco-Friendly Materials	Ensure the product meets health and environmental certifications to reduce environmental impact.

However, after converting the needs into design features, the research revealed conflicts between certain design features. For instance, there is a conflict between ergonomic design and multifunctional design: ergonomic design emphasizes comfort, while multifunctional design focuses on functional integration, which may affect comfort or visual coherence. To address these conflicts, the research team adjusted the priority of the design features to ensure a balance and operational feasibility, which will be further discussed in the analysis and discussion section.

#### 5.4.2 Design feature weight calculation.

**Fig 4:**
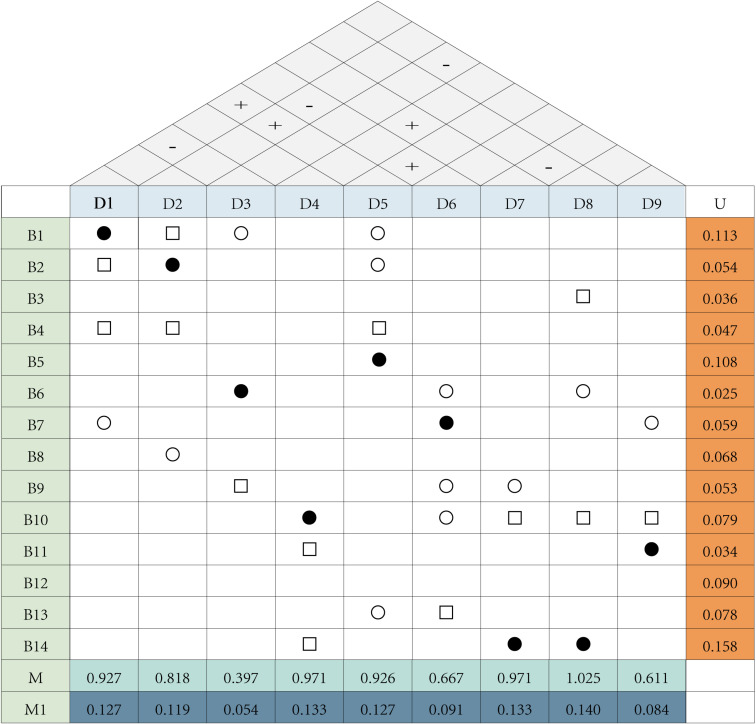
Constructing the house of quality. Note: Corresponds to the degree of relationship between user needs and design requirements, ● which represents the strong correlation, taking the value of 5; □ represents the medium correlation, taking the value of 3; ○ represents the weak correlation, taking the value of 1, and the two requirements have no correlation, then no marking, taking the value of 0. “**+”** indicates a positive correlation between design elements, and **“-”** indicates a negative correlation.

The core of QFD (Quality Function Deployment) is to construct a House of Quality. In this study, the House of Quality consists of five parts: 1. The top contains 9 main design features; 2. The left side lists 14 user needs; 3. The right side displays the weight values of user needs; 4. The bottom shows the importance ratings of design features; 5. The internal matrix is used to depict the relationship between user needs and design features. The degree of association between needs and design elements is determined through expert ratings, with scores classified as strong (5 points), moderate (3 points), and weak (1 point). The House of Quality clearly illustrates the connections between user needs and design features, lists the weights of design features, and helps identify positive and negative relationships among technologies, as shown in Fig 4.

To better understand the design process, this study uses U to represent the weight of user needs and M to represent the weight of design features, thereby establishing the House of Quality for user needs, as shown in Fig 4. In calculating the values for design feature M, ${\text{E}}_{\text{j}}$ represents the absolute importance weight of the design requirement, ${\text{e}}_{\text{j}}$ represents the relative importance weight, ${\text{w}}_{\text{i}}$ is the weight of the i-th user need, and ${\text{F}}_{\text{ij}}$ is the relationship value between the two.

The weighting formula for design requirements (M) is as follows (9):


$$\overset{\text{n}}{\underset{\text{i}=1}{\sum }}{\text{W}}_{\text{i}}\times {\text{F}}_{\text{ij}}\left(\text{i },\text{j}=1,2,\ldots ..,\text{n}\right)={\text{E}}_{\text{j}}$$ (9)

The formula for the relative importance weighting of design requirements is as (10):


$$\frac{{\text{E}}_{\text{j}}}{\sum_{\text{j}=1}^{\text{n}}{\text{E}}_{\text{j}}}\left(\text{j}=1,2,\ldots .,\text{n}\right)={\text{e}}_{\text{j}}$$ (10)

To gain a clearer understanding of the proportion of weight vectors for each design element, we normalize M to obtain M1. As shown in Formula (11), ${\text{X}}_{\text{i}}$ represents the i-th data point in the original data, $\overset{\text{n}}{\underset{\text{j}=1}{\sum }}\times \text{j}$ is the sum of all data points, and ${\text{X}}_{\text{i}}^{\text{'}}$ represents the normalized data.


$${\text{X}}_{\text{i}}^{\text{'}}=\frac{{\text{X}}_{\text{i}}}{\sum_{\text{j}=1}^{\text{n}}\times \text{j}}$$ (11)

The calculated design feature weights are ranked from highest to lowest as follows: D8 > D7 = D4 > D1 = D5 > D2 > D6 > D9 > D3, as shown in Fig 4. Among the 9 design elements at the top, they have interrelated effects, with “−” indicating a negative correlation and “+” indicating a positive correlation. These conflicting relationships will be analyzed and addressed in the discussion and analysis section.

#### 5.4.3 Design feature weight calculation.

In the design and development stage, it is not about satisfying all user design elements indiscriminately, but rather determining the number of elements to be developed based on practical considerations [78]. Therefore, it is necessary for us to select the design optimization points that best align with user expectations for improvement. When resources are limited, the Pareto Principle often helps designers prioritize the design elements that have the greatest impact on the project, rather than allocating time and resources evenly. In this study, we applied the Pareto Principle to extract the 9 design elements identified through the QFD House of Quality analysis [79]. Calculate the cumulative weights of each design element according to Formula (12).


$${\text{W}}_{\text{i}}=\overset{\text{i}}{\underset{\text{j}=1}{\sum }}{\text{w}}_{\text{j}}$$ (12)

Here, ${\text{w}}_{\text{i}}$represents the cumulative weight of the i-th design element, indicating the total contribution from the 1st to the i-th design element.

The Pareto Principle, also known as the 80/20 rule, generally states that 20% of key factors account for 80% of the results or impacts [80]. In the field of design and development, this means that a small number of design elements have the greatest impact on user experience. Therefore, we only need to focus on the top 80% of cumulative weight, concentrating efforts on the design elements that most significantly affect user experience, rather than distributing resources evenly across all design elements. The setting of the cumulative weight threshold T=80% is based on the goal of this study, which is to identify and optimize the most critical design elements to enhance user experience. This study aims to focus on the design features that have the greatest impact on user experience and the highest optimization potential. Therefore, we set the cumulative weight threshold to T=80%, which represents the total weight covered by this study and aligns with the strategy of concentrating efforts on optimizing design elements. Design elements that reach this threshold contribute 80% of the impact on user experience. Projects meeting the cumulative T value are obtained using Formula (13), with the results shown in Table 18.

**Table 18:** Cumulative weight values of design elements.

Design Elements	$W_{i}$	$W_{k}$
D8	14%	14%
D7	13.3%	27.3%
D4	13.3%	40.6%
D1	12.7%	53.3%
D5	12.7%	66%
D2	11.9%	77.9%
D6	9.1%	87%
D9	8.4%	95.4%
D3	5.4%	100.8%


$${\text{W}}_{\text{k}}\geq \text{T}$$ (13)

Based on the calculation results, the design and development should focus on the top seven design elements (D8, D7, D4, D1, D5, D2, D6). Particularly, the top-ranked elements D8 (multi-functional design), D7 (usability and convenience), and D4 (ergonomic design) are crucial for enhancing product innovation, user experience, and market demand. D8 can meet the diverse needs of different user groups; therefore, product development should explore how to integrate various functions into the product to provide higher usability value and broader market adaptability. Secondly, D7 requires designers to deeply understand the usage habits of target users during the development process, optimize product workflows, ensure that users can easily get started, and reduce learning costs, which is essential for improving user satisfaction and market competitiveness. D4 should be integrated throughout the entire design phase by analyzing users’ physiological needs to ensure the comfort and suitability of the product, thereby enhancing the long-term user experience. Based on these findings, further exploration will be conducted on innovative design for tourism-oriented rural cultural products to identify key design elements that can maximize the enhancement of cultural products, as discussed in Chapter 6. These design elements will provide guidance for future rural cultural projects, ensuring that innovative designs have both cultural depth and market relevance, thus achieving sustainable development goals.

## 6. Discussion and analysis

**Fig 5:**
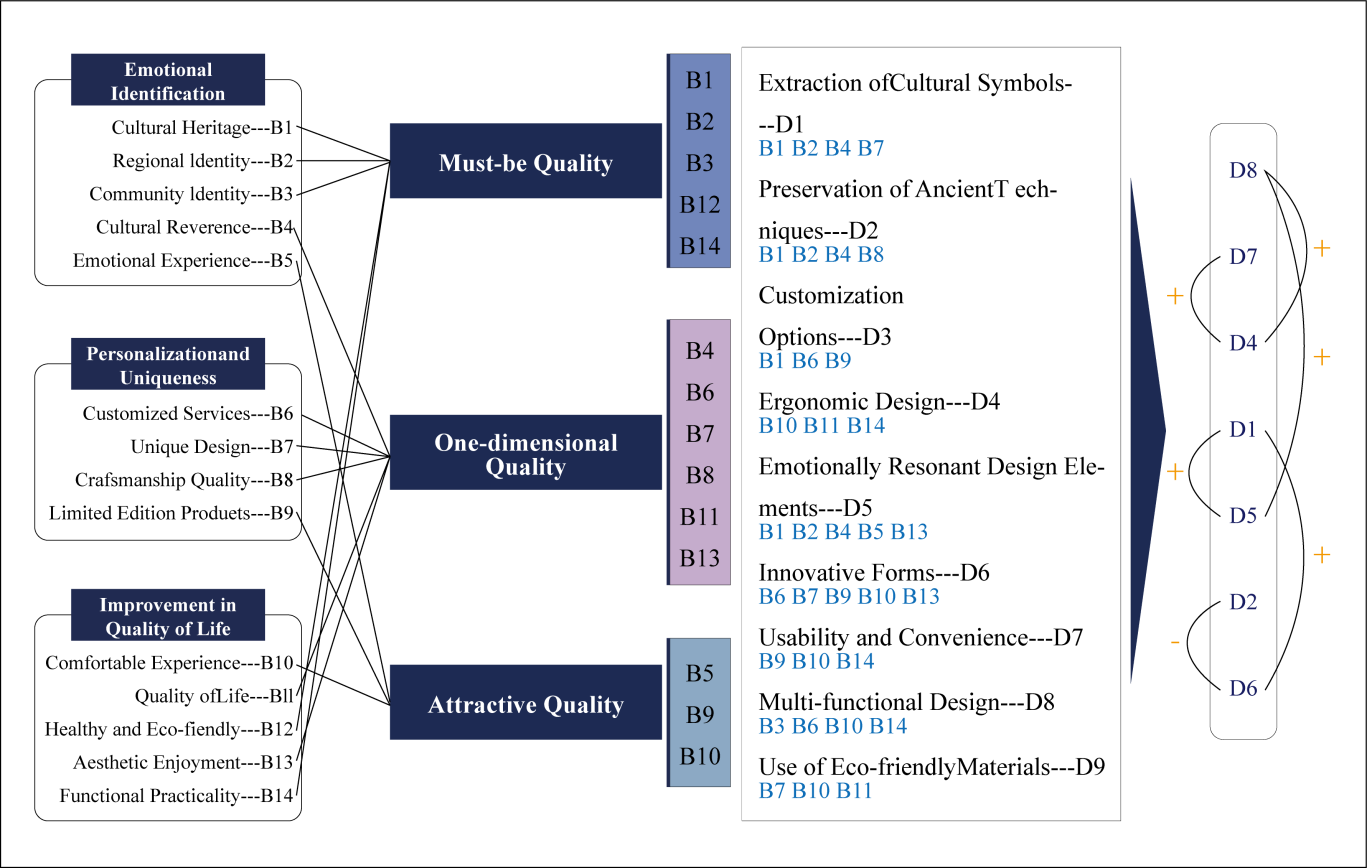
Research conclusion framework.

This study developed a research framework that transforms user needs into design elements, providing innovative ideas for the development of rural cultural creative products targeted at tourism in China. In the user needs exploration phase, grounded theory was first used to identify the three core user needs of the product: 1. emotional resonance, 2. individuality and uniqueness, 3. improvement of life quality. These needs stem from 14 major categories. Next, based on the KANO model, these needs were classified into basic needs, expected needs, and excitement needs, helping designers to clarify which needs must be met and which are potential improvement points that could enhance user satisfaction. In order to effectively transform these needs into design elements, the Analytic Hierarchy Process (AHP) was used to assign weights to the needs, providing a quantitative reference. Subsequently, the Quality Function Deployment (QFD) theory was applied to construct the House of Quality matrix, refining the 14 user needs into 9 key design elements and clarifying the relationships and priorities among them. The detailed research conclusion framework is shown in Fig 5. This process helped us identify which design elements most effectively meet user needs and revealed the interactions among these elements. It should be noted that these seven final design needs are interrelated, and describing a single design element in isolation may overlook its role in the overall design. To comprehensively understand the effectiveness of the design solution, we need to explore the interrelationship between design elements to ensure their combined impact is fully considered in the product design.

### 6.1 The synergistic effect of multi-functionality and ergonomics design

Multi-functionality design and ergonomics design elements show a positive correlation, exhibiting a synergistic effect in enhancing the product’s usability, comfort, aesthetic value, and market acceptance. In rural cultural creative products, the core idea of multi-functionality design lies in increasing the diversity and adaptability of the product to enhance its practicality while preserving its cultural heritage value. In contrast, ergonomics design emphasizes the product’s fit with the user’s body, aiming to provide a more comfortable user experience [81]. However, an excessive increase in functions may lead to operational complexity, thus reducing user comfort and efficiency [82]. Therefore, when designing multifunctional products, it must be combined with ergonomic principles to ensure that each function does not affect the overall comfort.

The advancement of Virtual Reality (VR) technology has made it an indispensable tool in product development, especially in function design verification and user experience testing [83]. Designers can use VR technology to create virtual models of products, conduct user interaction tests, observe user behaviors, and assess their emotional responses to product functions and cultural symbols. For example, in developing an intelligent tea set that incorporates traditional cultural elements, VR can simulate the user experience in different scenarios, helping designers optimize the functional layout and the presentation of cultural symbols. However, given the increasingly diversified user needs, it is unrealistic to incorporate all functions into a single product. Therefore, modular design emerges as a solution, allowing users to select the appropriate functional modules based on their needs, thus enhancing the product’s flexibility and market adaptability [84,85].

Moreover, to avoid operational complexity due to excessive functions, the Progressive Design strategy can be integrated with modular design [86]. In this strategy, the initial version of the product offers only basic functions, and more advanced functions are gradually unlocked as users become familiar with the product and their needs evolve. This approach not only avoids initial complexity but also allows the product to expand its functionality based on users’ actual habits and needs, providing a flexible user experience. The development of rural cultural creative products should carefully consider functional choices, combining them with ergonomic principles to avoid over-design. By complementing modular design and progressive design, the product’s market acceptance and user experience can be effectively enhanced, increasing its sales potential and market competitiveness.

### 6.2 Ease of use and intelligent design

The ease of use and convenience of a product are important standards for evaluating its design quality, and these two factors are closely related to ergonomics design, supporting each other. The core goal of ergonomics design is to ensure that the product fits the user’s body, thereby enhancing ease of use and convenience [87]. In the design of rural cultural creative products, the integration of these two factors can improve the product’s stability and operational convenience. For instance, designers can introduce adjustable components to provide more stable support in different usage scenarios. Additionally, the product’s operation should be as simplified as possible to increase convenience. This can be achieved by incorporating simple, intuitive operating mechanisms, such as replacing traditional screws and tools with latches or fasteners, or by using a “fold-unfold” design to avoid cumbersome assembly processes, thus reducing the user’s learning costs [88].

With the continuous advancement of technology, modern consumers’ demand for intelligent products is increasing [89]. Therefore, rural cultural creative products should not be limited to traditional design elements but should also integrate intelligent technologies and modern design thinking to better meet contemporary user needs. For example, an intelligent tea set system could incorporate built-in temperature sensors to automatically adjust the water temperature and provide personalized tea brewing suggestions based on user preferences. The integration of smart technology not only enhances the product’s convenience but also expands its functionality, allowing the product to adjust automatically based on real-time user needs or environmental conditions. This intelligent adjustment functionality can optimize the product’s performance according to users’ habits or environmental factors, further improving its applicability and user experience.

### 6.3 Cultural symbols and emotional design

The extraction of cultural symbols, emotional resonance design, and innovative forms are closely related design elements that collectively enhance the product’s cultural value and user experience. During the product development process, designers need to ensure that the extraction of cultural symbols and emotional resonance design are effectively integrated with modern functional needs. For instance, when designing tableware that incorporates traditional Chinese dragon patterns, the designer should retain the cultural significance of the dragon pattern while simplifying the lines and using modern design language to make it both culturally meaningful and fashionable. This approach satisfies consumers’ need for cultural heritage while enhancing the product’s market competitiveness. Specifically, the following aspects can be considered:

1. Extraction of Cultural Symbols: Designers should thoroughly study the local rural traditions and symbolic elements and combine systematic literature research [90], user feedback [91], and emotional testing [92] to select the cultural symbols with the greatest potential for emotional resonance. These symbols will become the core elements of the product design, but they need to be modernized through simplification and abstraction to ensure they balance cultural communication and modern aesthetics, thus meeting the demands of the contemporary market [93].
2. Emotional Resonance Design: Emotional resonance design aims to trigger users’ emotional responses through innovative applications of color, material, and form. Designers can use warm tones or natural materials to increase the product’s affinity [94]. To ensure the emotional resonance effect of the design, designers should validate whether the product effectively resonates with the target user group through field research and emotional testing.
3. Innovative Forms: Combining traditional cultural elements with modern design language is the key to achieving innovative forms. Designers should simplify complex patterns while retaining rural traditional motifs and use modern manufacturing processes for fine treatment to ensure the product retains cultural depth while meeting modern consumers’ usage habits and aesthetic standards. This way, the product can organically integrate tradition with modernity, offering both cultural richness and practicality as well as aesthetic appeal.

By appropriately utilizing these three design elements, rural cultural creative products can balance the inheritance of traditional culture with modern market needs, enhancing both user experience and market competitiveness.

Although this study provides a framework for capturing user needs and transforming them into design elements and discusses the topics of multi-functional design, ergonomics, ease of use, convenience, and cultural symbol extraction, the existing research is mainly based on static user need analysis and has not fully considered the dynamic nature of user needs over time and market conditions. Therefore, future research could focus on developing adaptive design systems that utilize real-time data and market feedback to adjust designs, incorporating lifecycle analysis and contextual sensing technology to ensure that the design can respond to changes in demand. Additionally, through market forecasting and agile design processes, designers can proactively anticipate and respond to future changes in demand. This approach can effectively enhance the flexibility and foresight of design. A continuous user feedback mechanism will also help designers capture demand changes in real-time, ensuring the long-term market competitiveness of products. These strategies will drive design towards a more flexible and forward-looking direction, ensuring its adaptation to the constantly changing market and user needs.

## 7. Conclusion

This study constructs a user-needs-based framework for rural cultural and creative product design, systematically exploring multifunctional design, ergonomics, usability, convenience, as well as the extraction of cultural symbols and emotional design elements. By analyzing the relationship between design elements and the transformation of user needs, the study expands the research perspective on rural cultural and creative product design and proposes multidimensional design principles. This framework not only provides theoretical support for the design of rural cultural products in China but also offers practical tools for global cultural product design. The innovation of this study lies in providing designers with a comprehensive analytical tool to help identify and meet user needs from diverse cultural backgrounds and market demands. Particularly in the context of increasing globalization and cultural diversity, designers and manufacturers can use this framework to develop innovative product strategies that align with different markets.

Based on the results of this study, we propose relevant suggestions for the development of rural cultural and creative products in China. From the perspective of market segmentation, the design of rural cultural and creative products should pay more attention to the differentiated needs of consumer groups, especially in terms of lifestyle and cultural background. Urban consumers tend to prefer smart, user-friendly products, while rural consumers place more emphasis on the use of traditional cultural symbols and natural materials. Therefore, designers should strike a balance between convenience, portability, and cultural depth, based on the different needs of consumer groups. For example, short-term tourists are more concerned with the portability and immediate usability of products, while long-term residents care more about the cultural value and long-term applicability of products. In the context of modern design, the integration of smart technologies is also crucial. Designers should consider how to incorporate smart adjustment functions into products to enhance adaptability and user experience. At the same time, designers should carefully balance traditional cultural symbols with innovative designs, avoiding over-reliance on traditional elements that may limit creativity. For users who prefer retro styles, designers can combine vintage elements with modern technology to meet emotional needs while ensuring the product’s modernity and practicality.

Although the design framework proposed in this study has practical significance, there are still some limitations. The extraction of cultural symbols is somewhat subjective, as the designer’s cultural background and experience may influence the interpretation of symbols, thus affecting market acceptance. Future research could reduce the subjectivity in symbol extraction by adopting a multicultural perspective and standardized tools, improving the universality of the design and its acceptance in global markets. Cross-cultural research and interviews would help to better understand the emotional value and cultural meaning of symbols, ensuring the accuracy of the design. In addition, this study did not fully account for the dynamic changes in user needs, especially in rapidly changing markets, where user needs may fluctuate significantly over time. Future research could use technologies like big data to track and analyze changes in user needs in real-time, providing more accurate user profiles and market trend predictions. By dynamically monitoring user behavior and feedback, designers can adjust product features promptly to adapt to the ever-changing market demands.

In summary, this study not only provides a systematic framework for the design of rural cultural and creative products in China but also offers methodological guidance for global cultural product design. Future research could further refine the framework by incorporating cross-cultural interpretations of cultural symbols and dynamic changes in user needs, thereby promoting innovation and development in the product design field and better meeting the diverse demands of the global market.

## Supporting information

S1 File Questionnaire interview outline. (DOCX)

S1 Checklist PLOSOne Human Subjects Research Checklist(1). (DOCX)
