# Supplementary material for: User-centric innovation strategies for cultural creative products in China’s rural tourism
Source: PLoS One. 2025 Apr 23;20(4):e0319474. doi: 10.1371/journal.pone.0319474 (PMC12017506; doi:10.1371/journal.pone.0319474)
Supplement: S1 File — (DOCX) [file pone.0319474.s001.docx]

The above is the interview outline for conducting semi-structured interviews with users, primarily exploring their demands for rural creative products. Semi-structured interviews are open-ended, allowing conversation to flow naturally based on the interviewee's interests and responses.

1. Name：
2. Age：

1. Gender：

1. Education：
2. Level Occupation：
3. Do you currently live in a rural or urban area? If urban, how often do you return to the countryside?
4. What types of cultural and creative products do you usually like to buy?
5. What factors are most important to you when purchasing cultural and creative products?
6. Do you prefer traditional or modern design when choosing cultural and creative products? Why?
7. What do you think are the unique attractions of rural cultural and creative products?
8. What is your main motivation for purchasing rural cultural and creative products?
9. What innovations do you want to see in the design of rural cultural and creative products?
10. What traditional elements do you think should be retained in the design of rural cultural and creative products?
11. What specific needs or expectations do you have for the functionality of rural cultural and creative products?
12. Are you willing to pay more for customized rural cultural and creative products? Why?
13. What are the main channels through which you purchase rural cultural and creative products?
14. When you buy rural cultural and creative products, do you pay attention to their production process or craftsmanship?
15. What do you think are the shortcomings in the experience of rural cultural and creative products in the current market?
16. What do you think is the future development trend of rural cultural and creative products?
17. Do you feel that rural cultural and creative products are now branded to what extent?
18. Do you think there is room for improvement in the promotion and publicity of rural cultural and creative products?
19. What advice do you have for rural cultural and creative product designers?
20. What do you think are some ways to better understand consumer needs and reflect them in product design?
21. Have you had any particular experiences with purchasing rural cultural and creative products? Can you share them?

**KANO Questionnaire Survey**

In order to design a rural cultural and creative products to meet the needs of users, I hope you can spare a few minutes to participate in the questionnaire, your valuable advice will be an important element of our product design improvement. The questionnaire adopts the scale type, from 5 to 1 satisfaction decreases, 5 is very satisfied, 4 is deserved, 3 is indifferent, 2 is barely accepted, 1 is very dissatisfied, a topic from the positive and negative sides to ask questions, thank you for your support!

1. Have you ever purchased any rural cultural and creative products?

Yes□ No□

2. What is your age?

18-20 years old□ 21-30 years old□ 31-40 years old□ 41-50 years old□ 51-60 years old□ 60 years old or older□ 3.

3. What is your education level?

Below high school□ High school and junior college□ Specialized□ Bachelor's degree□ Master's degree□ Doctoral degree and above□

4. Rural cultural and creative products have cultural heritage, your comment?

| 5. Very satisfied | 4. Deserves it | 3. Doesn't matter | 2. Barely accepts it | 1.Very dissatisfied |
| --- | --- | --- | --- | --- |
|  |  |  |  |  |

5. Rural cultural and creative products do not have cultural heritage, your comment?

| 5. Very satisfied | 4. Deserves it | 3. Doesn't matter | 2. Barely accepts it | 1.Very dissatisfied |
| --- | --- | --- | --- | --- |
|  |  |  |  |  |

6. Rural cultural and creative products are geographically attributed, your comments?

| 5. Very satisfied | 4. Deserves it | 3. Doesn't matter | 2. Barely accepts it | 1.Very dissatisfied |
| --- | --- | --- | --- | --- |
|  |  |  |  |  |

7. Rural cultural and creative products are not geographically attributable, your comment?

| 5. Very satisfied | 4. Deserves it | 3. Doesn't matter | 2. Barely accepts it | 1.Very dissatisfied |
| --- | --- | --- | --- | --- |
|  |  |  |  |  |

8. Rural cultural and creative products have social identity, your comment?

| 5. Very satisfied | 4. Deserves it | 3. Doesn't matter | 2. Barely accepts it | 1.Very dissatisfied |
| --- | --- | --- | --- | --- |
|  |  |  |  |  |

9. Rural cultural and creative products do not have social identity, your comment?

| 5. Very satisfied | 4. Deserves it | 3. Doesn't matter | 2. Barely accepts it | 1.Very dissatisfied |
| --- | --- | --- | --- | --- |
|  |  |  |  |  |

10. Rural cultural and creative products can inspire users' cultural reverence, your comment?

| 5. Very satisfied | 4. Deserves it | 3. Doesn't matter | 2. Barely accepts it | 1.Very dissatisfied |
| --- | --- | --- | --- | --- |
|  |  |  |  |  |

11. Rural cultural and creative products fail to inspire users' cultural reverence, your comment?

| 5. Very satisfied | 4. Deserves it | 3. Doesn't matter | 2. Barely accepts it | 1.Very dissatisfied |
| --- | --- | --- | --- | --- |
|  |  |  |  |  |

12.Rural cultural and creative products have a good emotional experience, your comment?

| 5. Very satisfied | 4. Deserves it | 3. Doesn't matter | 2. Barely accepts it | 1.Very dissatisfied |
| --- | --- | --- | --- | --- |
|  |  |  |  |  |

13. rural cultural and creative products do not have a good emotional experience, your comment?

| 5. Very satisfied | 4. Deserves it | 3. Doesn't matter | 2. Barely accepts it | 1.Very dissatisfied |
| --- | --- | --- | --- | --- |
|  |  |  |  |  |

14.Rural cultural and creative products have customized services, your comments?

| 5. Very satisfied | 4. Deserves it | 3. Doesn't matter | 2. Barely accepts it | 1.Very dissatisfied |
| --- | --- | --- | --- | --- |
|  |  |  |  |  |

15. Countryside Creative Products does not have a customization service, your comment?

| 5. Very satisfied | 4. Deserves it | 3. Doesn't matter | 2. Barely accepts it | 1.Very dissatisfied |
| --- | --- | --- | --- | --- |
|  |  |  |  |  |

16. Rural cultural and creative products with unique designs, your comments?

| 5. Very satisfied | 4. Deserves it | 3. Doesn't matter | 2. Barely accepts it | 1.Very dissatisfied |
| --- | --- | --- | --- | --- |
|  |  |  |  |  |

17. Rural cultural and creative products do not have a unique design, your comments?

| 5. Very satisfied | 4. Deserves it | 3. Doesn't matter | 2. Barely accepts it | 1.Very dissatisfied |
| --- | --- | --- | --- | --- |
|  |  |  |  |  |

18. Rural cultural and creative products with handicraft quality, your evaluation?

| 5. Very satisfied | 4. Deserves it | 3. Doesn't matter | 2. Barely accepts it | 1.Very dissatisfied |
| --- | --- | --- | --- | --- |
|  |  |  |  |  |

19. Rural cultural and creative products do not have the quality of handicrafts, your comment?

| 5. Very satisfied | 4. Deserves it | 3. Doesn't matter | 2. Barely accepts it | 1.Very dissatisfied |
| --- | --- | --- | --- | --- |
|  |  |  |  |  |

20. Rural cultural and creative products are limited edition products, your comments?

| 5. Very satisfied | 4. Deserves it | 3. Doesn't matter | 2. Barely accepts it | 1.Very dissatisfied |
| --- | --- | --- | --- | --- |
|  |  |  |  |  |

21. Rural cultural and creative products are not limited edition products, your comments?

| 5. Very satisfied | 4. Deserves it | 3. Doesn't matter | 2. Barely accepts it | 1.Very dissatisfied |
| --- | --- | --- | --- | --- |
|  |  |  |  |  |

22.Rural cultural and creative products have a cozy experience, your comment?

| 5. Very satisfied | 4. Deserves it | 3. Doesn't matter | 2. Barely accepts it | 1.Very dissatisfied |
| --- | --- | --- | --- | --- |
|  |  |  |  |  |

23. rural cultural and creative products do not have a comfortable experience, your comment?

| 5. Very satisfied | 4. Deserves it | 3. Doesn't matter | 2. Barely accepts it | 1.Very dissatisfied |
| --- | --- | --- | --- | --- |
|  |  |  |  |  |

24. Rural cultural and creative products can improve the quality of life, your comment?

| 5. Very satisfied | 4. Deserves it | 3. Doesn't matter | 2. Barely accepts it | 1.Very dissatisfied |
| --- | --- | --- | --- | --- |
|  |  |  |  |  |

25. Rural cultural and creative products can not improve the quality of life, your comments?

| 5. Very satisfied | 4. Deserves it | 3. Doesn't matter | 2. Barely accepts it | 1.Very dissatisfied |
| --- | --- | --- | --- | --- |
|  |  |  |  |  |

26. Rural cultural and creative products are healthy and environmentally friendly, your comments?

| 5. Very satisfied | 4. Deserves it | 3. Doesn't matter | 2. Barely accepts it | 1.Very dissatisfied |
| --- | --- | --- | --- | --- |
|  |  |  |  |  |

27. Rural cultural and creative products are not healthy and environmentally friendly, your comment?

| 5. Very satisfied | 4. Deserves it | 3. Doesn't matter | 2. Barely accepts it | 1.Very dissatisfied |
| --- | --- | --- | --- | --- |
|  |  |  |  |  |

28. Rural cultural and creative products have aesthetic enjoyment, your comment?

| 5. Very satisfied | 4. Deserves it | 3. Doesn't matter | 2. Barely accepts it | 1.Very dissatisfied |
| --- | --- | --- | --- | --- |
|  |  |  |  |  |

29. Rural cultural and creative products do not have aesthetic enjoyment, your comment?

| 5. Very satisfied | 4. Deserves it | 3. Doesn't matter | 2. Barely accepts it | 1.Very dissatisfied |
| --- | --- | --- | --- | --- |
|  |  |  |  |  |

30. Rural cultural and creative products have functional utility, your comment?

| 5. Very satisfied | 4. Deserves it | 3. Doesn't matter | 2. Barely accepts it | 1.Very dissatisfied |
| --- | --- | --- | --- | --- |
|  |  |  |  |  |

31. Rural cultural and creative products do not have functional utility, your comment?

| 5. Very satisfied | 4. Deserves it | 3. Doesn't matter | 2. Barely accepts it | 1.Very dissatisfied |
| --- | --- | --- | --- | --- |
|  |  |  |  |  |
